# Characterization of genome-wide ordered sequence-tagged *Mycobacterium* mutant libraries by Cartesian Pooling-Coordinate Sequencing

**DOI:** 10.1038/ncomms8106

**Published:** 2015-05-11

**Authors:** Kristof Vandewalle, Nele Festjens, Evelyn Plets, Marnik Vuylsteke, Yvan Saeys, Nico Callewaert

**Affiliations:** 1 Unit for Medical Biotechnology, Department of Medical Biotechnology Center (MBC), VIB, Technologiepark 927, Ghent B-9052, Belgium; 2 Laboratory for Protein Biochemistry and Biomolecular Engineering, Department of Biochemistry and Microbiology, Ghent University, Ledeganckstraat 35, Ghent B-9000, Belgium; 3 Gnomixx, Onafhankelijkheidslaan 38, Ghent B-9000, Belgium; 4 Unit for Immunoregulation and Mucosal Immunology, Inflammation Research Center (IRC), VIB, Technologiepark 927, Ghent B-9052, Belgium

## Abstract

Reverse genetics research approaches require the availability of methods to rapidly generate specific mutants. Alternatively, where these methods are lacking, the construction of pre-characterized libraries of mutants can be extremely valuable. However, this can be complex, expensive and time consuming. Here, we describe a robust, easy to implement parallel sequencing-based method (Cartesian Pooling-Coordinate Sequencing or CP-CSeq) that reports both on the identity as well as on the location of sequence-tagged biological entities in well-plate archived clone collections. We demonstrate this approach using a transposon insertion mutant library of the *Mycobacterium bovis* BCG vaccine strain, providing the largest resource of mutants in any strain of the *M. tuberculosis* complex. The method is applicable to any entity for which sequence-tagged identification is possible.

## Abstract (web summary)

The generation of characterized panels of specific mutants is an essential but time-consuming step of reverse genetic studies. Here Vandewalle *et al*. describe CP-CSeq, an easy to implement parallel sequencing method for rapid library construction.

## Graphical abstract

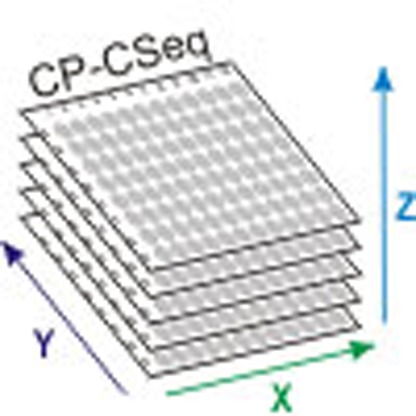

Geneticists often make use of large bulk libraries of sequence-tagged biological entities such as mutants or cloned DNA. These are highly valuable tools, for example, for phenotypic screens^1^ or regulatory network studies^2^. Many techniques are available to generate such libraries with many thousands of clones (for example, through mutagenesis), which are thus obtained as a very complex mixture. With such mixed-clone libraries, scientists are mainly limited to experiments where these clones can be screened in bulk^3^. In contrast, the majority of hypothesis-driven research requires the availability of individual, characterized clones (that is, single cDNA's, single mutants, single shRNA's and so on). Such archived single-clone libraries are much more difficult and costly to generate and to characterize the identity of each clone. An example can be found in tuberculosis research, where mutants in genes of interest are among others required for the study of potential drug targets and for live attenuated vaccine engineering^4^,^5^. Owing to the slow growth of Mycobacteria and limited effectiveness of classical techniques of gene inactivation, pre-characterized genome-wide mutant resources would be highly valuable^6^.

Mycobacteriophage-delivered transposon (Tn) mutagenesis is well established as an efficient tagged mutagenesis approach in Mycobacteria^7^. However, it yields a complex mixture of tens of thousands of strains, each with a Mariner Tn insertion in a TA dinucleotide sequence. Such mutant mixtures are useful for analysis of competitive mutant behaviour in bulk^8^, but not for investigations in which a single mutant or a set of such mutants in defined genes is needed. Ordered Tn insertion libraries can be picked from such Tn mutant mixtures and PCR-screened for insertions in a locus of interest^9^, but this procedure needs to be repeated for every gene and is prone to failure due to the high GC content of these genomes. Alternatively, library characterization through clone-by-clone sequencing of DNA regions flanking the Tn has been reported, but is obviously very laborious^10^. To our knowledge, the largest characterized Tn insertion resources in *Mycobacterium tuberculosis* (*M. tb*) contain no more than 1,329 (ref. 11) and 1,403 (ref. 12) clones. These and other mutants were integrated by the Tuberculosis TARGET programme, now offering over 4,300 defined Tn insertion mutants in *M. tb*^13^.

We have aimed at developing an easy-to-implement, cost- and time-effective, massively parallel sequencing-based approach (called Cartesian Pooling-Coordinate Sequencing or CP-CSeq) to deal with the characterization of such large collections of sequence-tagged clones. We illustrate it here for a large 9,216 clone ordered Tn insertion mutant library of the vaccine strain *M. bovis* Bacillus Calmette–Guérin (BCG). Our method first entails the pooling of the entire library along its Cartesian coordinates (*X*, *Y* and *Z*) to downscale the number of samples that need to be processed (*n*=40). Then, a custom Illumina library preparation protocol on these Cartesian pools enriches for the Tn-flanking genomic regions and adds pool-specific barcodes for multiplexed deep sequencing. Following sequencing of this mix of barcoded samples and mutant deconvolution, we obtained a resource of *M. bovis* BCG mutants in which 64% of the non-essential *M. tb* orthologues are disrupted. We demonstrate how this approach strongly contributes towards the endeavour of generating a mutant for every gene in the genome of such slow-growing Mycobacteria, which constitute some of the most important pathogens of mankind.

## Results

### Cartesian pooling concept

**Figure 1:**
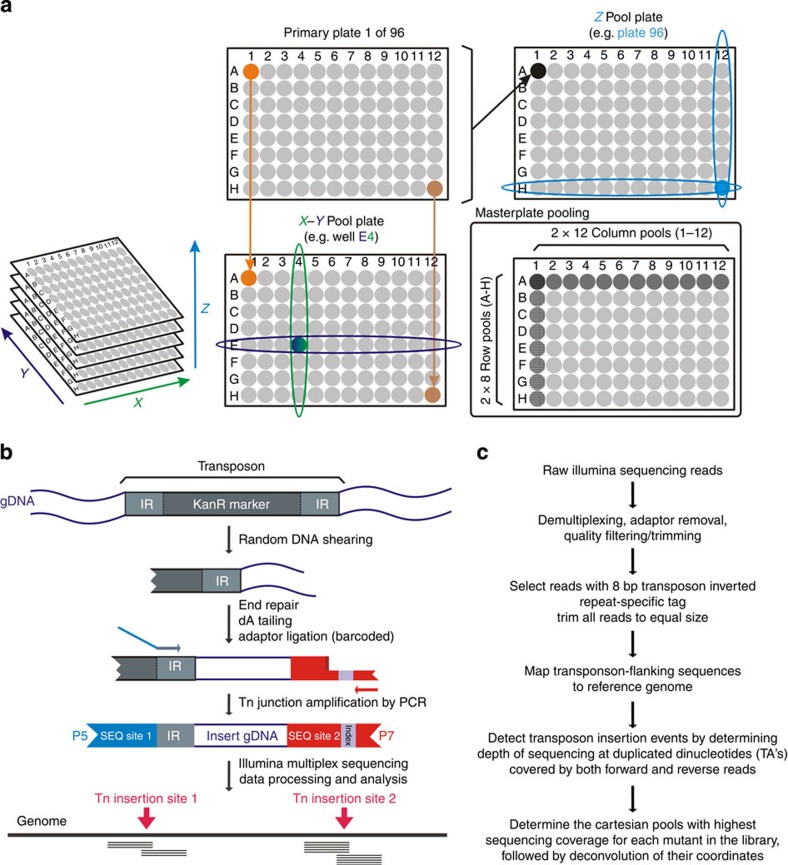
Cartesian Pooling-Coordinate Sequencing (CP-CSeq) concept for simultaneously determining both tag sequence and library coordinates of the biological entity containing the tag sequence. Here, the optimized concept is presented in the case of *M. bovis* BCG transposon-tagged mutants. (**a**) Layout and Cartesian Pooling of a 96 × 96-well library of sequence-tagged biological entities (for example, *M. bovis* BCG transposon insertion mutants). Each entity's position in the library is characterized by three Cartesian coordinates (*X*, *Y* and *Z*). To create the *X*–*Y* Pool Plate, a small culture volume of one specific well position (for example, A1) in all of the 96-well plates was transferred to and thus pooled in the same specific well (for example, A1) of the masterplate, thus keeping the respective positions within each primary plate (*X* and *Y* coordinates). Subsequently, the *Z* pool plate was prepared by pooling all of the 96 wells of a primary plate in one single well of the masterplate (*Z*-coordinate). Next, each row and each column of both masterplates were pooled in column (*n*=12) and row (*n*=8) pools, giving a total of 40 samples that represent the 96 × 96 clone library. (**b**) Coordinate-Seq sequencing library preparation links a pool-specific barcode to the sequence tag that identifies each biological entity in each pool. Here, the protocol is optimized for Himar1 transposon-flanking sequence tags. (**c**) Coordinate-Seq sequence data processing for transposon insertion mutants.

Upon mycobacteriophage-based Himar 1 Tn mutagenesis^14^, 9,216 (96 × 96-well plates) clones were picked to create an ordered library^9^. The location of each mutant in the archived library is characterized by three Cartesian coordinates (*X*, *Y* and *Z*). The *X* and *Y* coordinates pinpoint a mutant to a well position in the ordered 96-well plate stack, whereas the *Z* coordinate determines in which particular plate in the stack a clone is situated (Fig. 1a). We devised a novel pooling strategy along the library's Cartesian coordinates to downscale the number of samples to be processed. To create a first pooled masterplate, a small culture volume of one specific well position (for example, A1) in all of the 96-well plates was transferred to and thus pooled in the same specific well (for example, A1) of the masterplate, thus keeping the respective positions within each primary plate (*X* and *Y* coordinates). Subsequently, a second masterplate was prepared by pooling all of the 96 wells of a primary plate in one single well of the masterplate (*Z* coordinate; Fig. 1a). Next, each row and each column of both masterplates were pooled in column (*n*=12) and row (*n*=8) pools, giving a total of 40 samples that represent the 96 × 96 clone library (Fig. 1a, for detailed practical implementations using different types of multichannel pipetting devices, see Supplementary Methods). This pooling strategy thus captures the positional information of the 9,216 mutants in just 40 samples. In the perfect case, each mutant is present in 4 out of 40 pools (one column and one row pool of both masterplates), and from the identity of these 4 master pools, the original position of this clone in the library can be exactly determined. At this point, the problem is reduced to introducing barcode sequences identifying the specific master pools, adjoining the Tn-flanking DNA sequences that are generated from them.

### Library preparation and mutant deconvolution

The sequencing library preparation method is outlined in Fig. 1b. Briefly, genomic DNA of each of the 40 pools was fragmented and ligated to customized adaptors, containing different unique barcodes for multiplex sequencing. Next, the Tn junctions were enriched by PCR amplification with primers hybridizing to the Tn ends and to the adaptor. The PCR products were then combined and size selected before sequencing on an Illumina chip. This approach was rigorously optimized during proof-of-concept experiments using a subsection of the library, as discussed in more detail in Supplementary Note 1.

Following sequencing of the mix of barcoded Tn-flanking sequence samples derived from all 40 pools on a single lane of an Illumina HiSeq2500 chip, the reads were demultiplexed and quality filtered (Fig. 1c). After removal of the adaptor sequences, reads that were lacking the Tn-specific tag were excluded from the data set. The remaining reads were trimmed to equal size (25 bp) and mapped to the *M. bovis* BCG Pasteur reference genome. This sequencing read size trimming was important to avoid coverage calculation errors at Tn insertion sites that are very close together in the genome. To determine the exact position of each mutant within the 96 × 96-well library, we calculated the read coverage at all possible TA insertion sites within each of the 40 pools. Only reads that map with their 5′ end at these potential TA insertion sites were considered for this coverage calculation.

### *Mycobacterium bovis* BCG mutant resource

**Figure 2:**
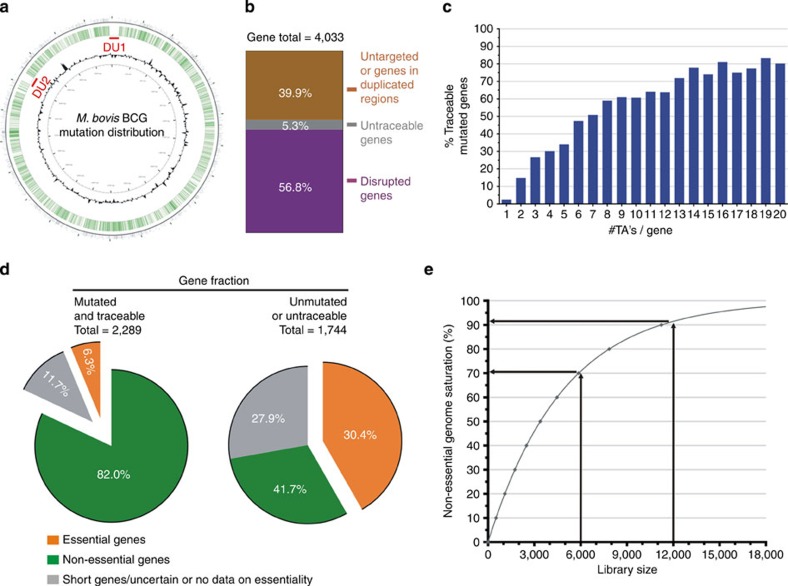
Results of Cartesian Pooling-Coordinate Sequencing on a 96 × 96-well transposon insertion library of *M. bovis* BCG. (**a**) Visualization of the *M. bovis* BCG Pasteur reference genome. GC% (black) and Tn insertions that were identified and coordinate-determined in our 96 × 96-well mutant library (green bars). Duplicated regions of the genome are marked in red (DU1 and DU2). (**b**) Distribution between disrupted ORFs for which the location in the library of at least one disruption mutant is known (purple), disrupted ORFs for which the locations of their disruption mutants are unknown (‘untraceable'; grey) and untargeted ORFs (brown). (**c**) Relation between the fraction and the number of TA's in the ORF. Genes for which the orthologues are known to be essential in *M. tb* are omitted from the analysis. (**d**) Gene distribution according to gene-essentiality of *M. tb* orthologues of both the mutated and unmutated/untraceable gene fraction in our *M. bovis* BCG Tn insertion library. Genes for which no reliable data are available with regard to their essentiality for *in vitro* growth are shown in grey. (**e**) Theoretical model (based on the ‘coupon collector's' problem) to estimate the library size needed to target a certain percentage of the non-essential genome with at least one disruption every 12 TA's (=median number of TA's per gene in the *M. bovis* BCG Pasteur genome). The model predicts that with 6,072 mutants, 71% of the non-essential genes should be hit at least once, which is slightly above what we observed in our data set (64%). Doubling the amount of mutants will allow disrupting almost all targetable genes in this genome.

This integrated analytical approach of CP-CSeq provided evidence for Tn insertions at 8,259 sites, with a further 542 at sites in the genome where reads cannot be uniquely mapped (i.e. duplicated regions and highly repetitive genes; Fig. 2a and Supplementary Data 1). Over 77% (6,383) of the 8,259 uniquely mapped insertion events had sufficient sequencing coverage in the expected number of pools (4) for customized software-based automated identification of the mutant's position in the archived clone library. About 84% of these insertions map within the ORF of known genes, targeting 57% (2,289/4,033) of the total number of genes in the BCG genome (Fig. 2b) and 64% (2,146/3,359) of the genes of which the *M. tb* orthologues are not essential for *in vitro* growth^15^,^16^. The remaining genes that we did not hit largely contain few TAs (Fig. 2c), or are genes for which the essentiality is unsure in *M. tb* (Fig. 2d).

**Figure 3:**
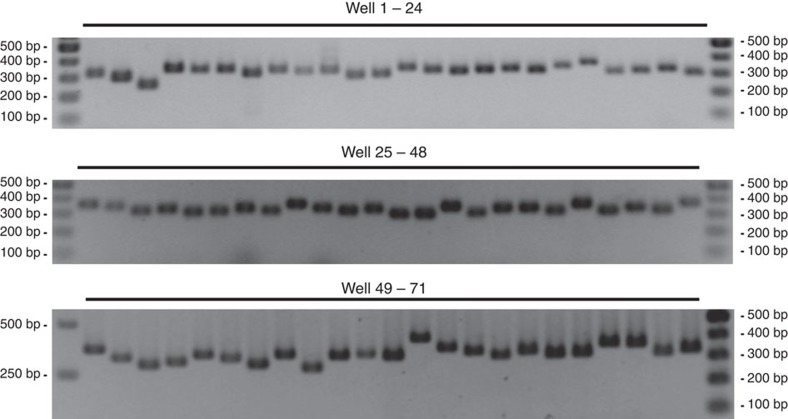
Positional validation of a subset of Tn insertion mutants by PCR. The CP-CSeq algorithm assigned 71 transposon insertion mutants to library plate 92. These assignments were verified by PCR with a primer hybridizing to the transposon inverted repeat and a gene specific primer. All CP-CSeq assignments were confirmed. The list of mutants and their corresponding primers can be found in Supplementary Data 2.

**Figure 4:**
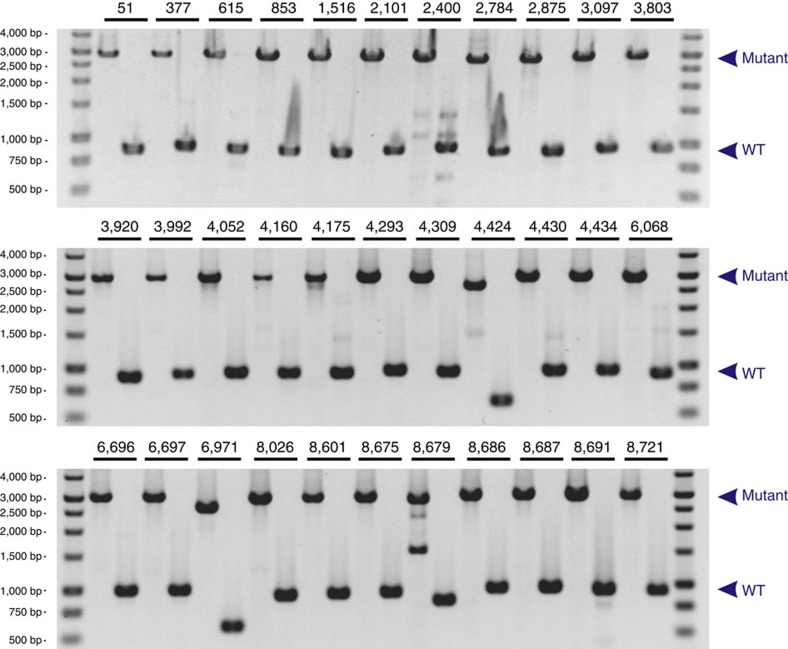
Verification by PCR of CP-CSeq assignment and of clonal purity of a subset of Tn insertion mutants, following single-clone picking from library glycerol stocks. The numbers correspond to the unique mutant ID in Supplementary Data 1 (TnInsertion-x) and a unique primer set for each mutant (Supplementary Data 2). These primers hybridize up- and downstream of the investigated transposon insertion event. PCR reactions were done both on mutant (first lane) and wild-type (WT; second lane) genomic DNA for all primer sets.

By PCR analysis, we verified the presence of all of the 71 particular mutants that were assigned by our algorithm to a particular 96-well plate (plate 92; Fig. 3). We further confirmed the position of another 33 mutants that were assigned by CP-CSeq to positions across the library (Fig. 4). 100% of all these CP-CSeq assignments were correct. Picking five to ten clones seeded from the glycerol stocks of the mutants was sufficient to recover the mutant in a clonally pure form. Such passing over a single-clone isolation step is a common good microbiology practice, and also necessary here, as some frequency of picking multiple clones to the same well is unavoidable during ordered library preparation because of the strong clumping behaviour of Mycobacteria of the *M. tb* complex. Moreover, some level of well-to-well cross-contamination is also to be expected during cultivation and library handling, especially as these organisms are highly adapted to airborne transmission. Clearly, our methodology is sufficiently robust to such unavoidable experimental complexities, strongly enhancing its real-life utility.

To benchmark these results to the theoretically expected number of targeted genes, we modelled the relation between the library size (number of uniquely mapped Tn insertions) and the percentage of non-essential genomic regions that are likely to get targeted by Himar1 Tn mutagenesis. This statistical problem is similar to the ‘coupon collector's' problem^17^. First, we calculated from the *M. bovis* BCG Pasteur genome that the median number of TA sites per non-essential-coding sequence is 12. Using this number, we obtained the model illustrated in Fig. 2e, which predicts that a library such as ours of 6,072 traceable Tn insertion mutants (311 insertions in essential *M. tb* orthologues excluded) should target 71% of the non-essential genes, which is close to the observed number (±64%). The difference is at least partially due to the presence in the genome of duplicated and repetitive regions in which mutants cannot be mapped using CP-CSeq. The model furthermore forecasts that performing the experiment twice to double the number of traceable Tn insertions is expected to hit up to 90% of the targetable genes, coming close to a full mutant resource for these organisms.

## Discussion

Our data demonstrate that this straightforward CP-CSeq method is highly competitive with much more complicated methods that were previously developed for similar purposes. Goodman *et al*. provided a complex protocol for characterizing clonally arrayed Tn insertion libraries using a liquid-handling robot to distribute archived mutant *B. thetaiotaomicron* strains across a subset of pools, according to a specific binary pattern^18^. However, tracing identical mutants to their specific locations in the library remained difficult. To diminish this issue, Erlich *et al*. proposed DNA Sudoku^19^. Although this allowed for an overall high accuracy in the analysed plasmid libraries, the pooling approach remains extremely complicated, yielding hundreds of pooled samples to be processed. Moreover, these methods are heavily dependent on complex programming of robotic equipment, a skill and resource not readily accessible for many scientists. This is in contrast to CP-CSeq, as sample transfer steps along Cartesian coordinates are highly intuitive and are the most common operational mode of liquid/clone-handling robots. It may be of note that we pooled the *M. bovis* BCG library completely through manual pipetting, requiring the efforts of two team members for just a few days. The method is thus accessible to any laboratory, including those without robots.

Our CP-CSeq method already provided the largest resource of mutants in any strain of the *M. tuberculosis* complex reported so far, and allows to create an almost comprehensive set of genome-wide mutant strains in less than the time required to generate even a single mutant with classical techniques^6^. Next to Tn mutagenesis as used here, recent advances in recombineering and phage transduction in Mycobacteria will likely increase the versatility and precision of sequence-tagged mutagenesis in these organisms^20^. Rather than applying these new methods in a laborious and costly gene-by-gene approach as currently attempted^20^, there is now scope for single-step genome-wide bulk recombineering in all target genes, followed by deconvolution using CP-CSeq. These innovations will dramatically speed up hypothesis testing in *Mycobacterium* biology.

Similar issues with difficult genetics and/or slow growth are prevalent throughout biology, as is the need to cost- and time-effectively characterize ordered libraries of biological entities (such as plasmid inserts of DNA libraries). The CP-CSeq concept and methodology should be broadly portable to such other settings.

## Methods

### Strains and media

The streptomycin-resistant *M. bovis* BCG strain 1721 (*RpsL*, K43R; a gift of Dr P. Sander, Institute for Medical Microbiology, Zurich) and its mutants were grown in Middlebrook 7H9 broth (Difco) supplemented with 0.05% Tween-80, Middlebrook OADC (Becton Dickinson) and appropriate antibiotic selection (100 μg per ml streptomycin for wild type and additionally 50 μg per ml kanamycin for the mutants) when grown in liquid culture. Difco Middlebrook 7H10 agar (similarly supplemented) was used for growth on solid culture. We selected the mutants on 7H10 plates supplemented with 20 μg per ml kanamycin. For the propagation of the phage, a streptomycin-resistant *M. smegmatis* strain (also obtained from Dr P. Sander) was used and grown in the Middlebrook 7H9 broth (Difco) supplemented with Middlebrook ADC (Becton Dickinson) without Tween-80 or antibiotics. Library cultures were cultivated in 96-well U-bottom tissue culture plates with low evaporation lid (Falcon; 200 μl cultures). Individual clones were grown in static or shaking culture flasks.

### Construction and Cartesian pooling of the mutant library

The Tn donor phagemid, ΦmycomarT7 (received as a gift from Prof Dr Eric Rubin, Harvard School of Public Health, Boston), was propagated in *M. smegmatis* to generate phage stocks. In short, *M. smegmatis* cultures (grown in 7H9 supplemented with ADC and Tween-80 until an OD_600_=1.0) were washed three times with MP buffer (50 mM of Tris, pH 7.5, 150 mM of NaCl, 10 mM of MgSO_4_ and 2 mM of CaCl_2_) and resuspended in an equal volume of MP buffer. Serial dilutions of the ΦmycomarT7 phage were made in MP buffer, added to 200 μl *M. smegmatis* aliquots and 3 ml of top agar (4.7 g l^−1^ Middlebrook 7H9broth base (Difco), 7 g l^−1^ Bacto-agar (Difco), 0.1% glucose, 0.1 mM of CaCl_2_; autoclaved and cooled to 42 °C) and spread out on 7H10 plates (supplemented with ADC). After incubation for 2 days at 30 °C, plates containing confluent plaques were incubated for 4 h with MP buffer at 4 °C. The phage-containing mixtures were then collected, filtered (0.2 μm filter) and stored at 4 °C until further use^21^.

*M. bovis* BCG, grown to OD_600_=1.0, was transduced as follows. In short, the cells (corresponding to 50 ml of culture volume grown to OD_600_=1.0) were washed three times with MP buffer and resuspended in 5 ml of MP buffer. The cells were then infected with 10^10^ p.f.u. (plaque-forming units) of ΦmycomarT7 phage for 3 h at 37 °C (ref. 15).The library was plated on 7H10 agar plates containing 50 μg per ml kanamycin and 100 μg per ml streptomycin, as the Tn contains the kanamycin-resistant gene. The plating was performed at the appropriate dilution to obtain well-separated single colonies. Once the colonies were well grown on the 7H10 agar plates, they were manually picked using sterile toothpicks into 96-well plates, each plate containing 96 individual mutants (a total of 9,216 clones). Care was taken to avoid clustered colonies, which are very prevalent due to the clumping behaviour of *M. bovis* BCG. Individual mutants were grown in 96-well plates in 200 μl of Middlebrook 7H9 broth supplemented with 10% OADC and 50 μg per ml kanamycin and 100 μg per ml streptomycin for 21 days and aliquots were frozen in 20% glycerol at −80 °C. A detailed procedure of how we constructed the library can be found in Supplementary Methods, section 1.

For the Cartesian pooling concept, 200 μl library cultures (96 × 96-well plates) were grown for 21 days and processed in four sets of 24 plates as follows. The position of each mutant, one per well, is characterized by three coordinates (*X*, *Y* and *Z*). First, cultures of each well (70 μl aliquots) were transferred into their respective wells in an *X*–*Y* Pool Plate (96-deep-well plate), thus keeping the respective positions within each primary plate (*X* and *Y* coordinates). Then, a second batch of each culture (70 μl aliquots) was transferred into a second deep well plate (2 Pool Plate), where each well collects the samples of an entire primary plate (*Z* coordinate). This was done for all four sets of 24 plates. Next, for each submasterplate, all rows and columns were pooled into their respective column (900 μl aliquots) and row (600 μl aliquots) subpools. Finally, the corresponding pools of the four subsets were combined, leading to a total of 40 pooled samples. A detailed practical implementation using different types of multichannel pipetting devices can be found in Supplementary Methods, section 2.

### Illumina Coordinate-Seq library preparation

The optimization work for the library preparation method is described in Supplementary Note 1. All primer and adaptor sequences are listed in Supplementary Data 2. Genomic DNA of the Mycobacterial pools was prepared as described in Supplementary Methods^22^.

Genomic DNA (±1 μg) derived from the 40 mutant pools of the complete library was sonicated (Bioruptor standard, Diagenode), blunt-end repaired and A tailed (NEBNext end-repair and dA-tailing module, NEB). At several steps in the protocol, DNA fragments were each purified with Agencourt AMPure XP beads (Beckman Coulter). These fragments were ligated (FastLigase, Enzymatics) to custom-made adaptors (IDT DNA), containing a T overhang, the standard Illumina index sequencing site and P7 sequence, and a different 8 bp barcode for each individual pool. Subsequently, a nested PCR was performed using primers P7 (complementary to the Illumina P7 sequence) and a mix of primers P5-IR2a-d (hybridizes with the Tn inverted repeat and contains the standard Illumina P5 and forward sequencing primer sequence). The resulting PCR products were combined, size selected (±500 bp), purified and sequenced with the standard sequencing primers on 1 lane of an Illumina HiSeq2,500 chip, generating 71,464,831 single-end sequencing reads of 100 bp. A detailed procedure of the library preparation protocol can be found in Supplementary Methods, section 3.

### Sequencing data analysis

The raw Illumina sequencing data were processed with CLC Genomics Workbench (http://www.clcbio.com/products/clc-genomics-workbench/) and the open source Galaxy platform (http://galaxyproject.org/). After Illumina adaptor and read quality-based trimming, the sequence reads were parsed for 100% identity to the 8-bp specific Tn inverted repeat tag. Matching reads were stripped from this tag, trimmed to equal size (25 and 60 bp) and mapped to the *M. bovis* BCG Pasteur reference genome (strain 1173P2, Genbank accession number AM408590.1). Sequence reads that aligned to more than one position on the genome (duplicated or repetitive regions) were not further considered. True Tn insertion events were identified by only considering dinucleotides where both forward and reverse reads mapped, overlapping at their 5′ end on that particular dinucleotide^8^. The coverage of such reads was calculated in each of the 40 pools and used to determine each mutant's location, if possible. First, to avoid mutants with one or more missing coordinates, Tn insertion locations with an overall read count below 300 were not considered. Then, for each remaining Tn inserted dinucleotide, mutant pools with very low sequencing coverage (<15 reads) were ignored. For cases where a Tn insertion appeared to be present several times in the library, a heuristic was applied to find the location with most experimental evidence. To this end, the mutant pools were sorted according to the number of reads mapping to the dinucleotide of interest. The pool with the highest read count was used to map the Tn to a unique location if that read count was substantially higher than the second highest read count (1.5 times higher for read counts >1,000 and three times higher for reads counts <1,000). A detailed procedure to perform the sequencing data analysis can be found in Supplementary Methods, section 4.

### Verification of the Tn insertion sites by PCR

Plate 92 of the library was grown in 7H9 medium, supplemented with OADC, in 96-well U-bottom tissue culture plates. After 3 weeks, these cultures (100 μl aliquots) were heated to 98 °C for 30 min to release genomic DNA in the medium. After centrifugation, the supernatants were used in a PCR reaction with a primer hybridizing to the Tn inverted repeat (TL006) and gene-specific primers hybridizing 200–400 bp upstream (Supplementary Data 2).

Another subset of 33 Tn insertion mutants were streaked on 7H10 plates supplemented with 50 μg per ml kanamycine. After 2–4 weeks, single colonies were inoculated in liquid 7H9 medium. Cultures were grown to early stationary phase and then used for genomic DNA preparation. This genomic DNA was used as template in a PCR reaction (Phusion polymerase, Finnzymes) with primers±500 bp up- and downstream of the predicted Tn insertion site (Supplementary Data 2). Amplification of the wild-type gene results in a band of 500–1,000 bp, the Tn-disrupted amplicon is 2 kb larger.

PCR conditions were as follows: 98 °C for 3 min; 30 cycles of denaturation (98 °C for 20 s), annealing and extension (72 °C for 2 min); 72 °C for 5 min. PCR products were run on a 1.2% agarose gel, stained with ethidiumbromide and visualized under ultraviolet light. Scans of the entire gels can be found in the Supplementary Information file (Supplementary Figs 1 and 2).

### Library size calculations

To model the library size needed to hit increasing fractions of the genome with at least one Tn, we took a theoretical approach by considering this problem similar to the ‘coupon collector's problem'. The number of non-essential possible insertion sites is 58,815. This was calculated by subtracting the number of TA's in ‘essential' (*M. tb*)^16^ genes (14,355) from the total number of TA dinucleotides present in the genome (72,870). The number of 58,815 TA's in non-essential genomic regions was cut in sections of a specified number of TA's. As the median number of TA's in ‘non-essential' *M. bovis* BCG genes is 12, we chose this number. In this way, we modelled that the genome consists of 58,815/12=4,876 non-essential genome regions (‘genes'), each containing 12 TA's. To calculate the expected library size E(T) needed to target all of these genes, we used the ‘coupon collector's' expression E(T)=*n*. (*n*^−1^+…+½+1)^17^, where *n* is the number of non-essential genes (as modelled by a genomic stretch containing 12 consecutive TA's). Library sizes expected to target 10–90% of the genes (in steps of 10%) were calculated and plotted using Graphpad.

## Author contributions

K.V. designed and performed the experiments, analysed the data and co-wrote the manuscript. N.F. and E.P. aided in growing and pooling the transposon insertion mutant library. N.F. assisted in experimental design and interpretation, and carefully revised the manuscript. M.V. assisted with the library size calculations. Y.S. wrote the BioPerl algorithm for automated location assignment. N.C. conceived the pooling approach, initiated the project, assisted in experimental design and interpretation and co-wrote the manuscript.

## Additional information

**Accession codes:** The novel sequencing data generated in this study has been deposited at NCBI's Sequence Read Archive under project code SRP056542.

**How to cite this article:** Vandewalle, K. *et al*. Characterization of genome-wide ordered sequence-tagged *Mycobacterium* mutant libraries by Cartesian Pooling-Coordinate Sequencing. *Nat. Commun*. 6:7106 doi: 10.1038/ncomms8106 (2015).

## Supplementary Material

Supplementary Information Supplementary Figures 1-6, Supplementary Note 1, Supplementary Methods and Supplementary References

Supplementary Data 1 List containing all transposon insertion events in our *M. bovis* BCG mutant library.

Supplementary Data 2 List containing all primers and adaptors used in this study.

Supplementary Data 3 Galaxy workflows and BioPerl algorithm that were used to deconvolute the mutant positions.
