# Supplementary material for: Characterization of genome-wide ordered sequence-tagged Mycobacterium mutant libraries by Cartesian Pooling-Coordinate Sequencing
Source: Nat Commun. 2015 May 11;6:7106. doi: 10.1038/ncomms8106 (PMC4432585; doi:10.1038/ncomms8106)
Supplement: Supplementary Information — Supplementary Figures 1-6, Supplementary Note 1, Supplementary Methods and Supplementary References [file ncomms8106-s1.pdf]

Supplementary Information

Supplementary Figures

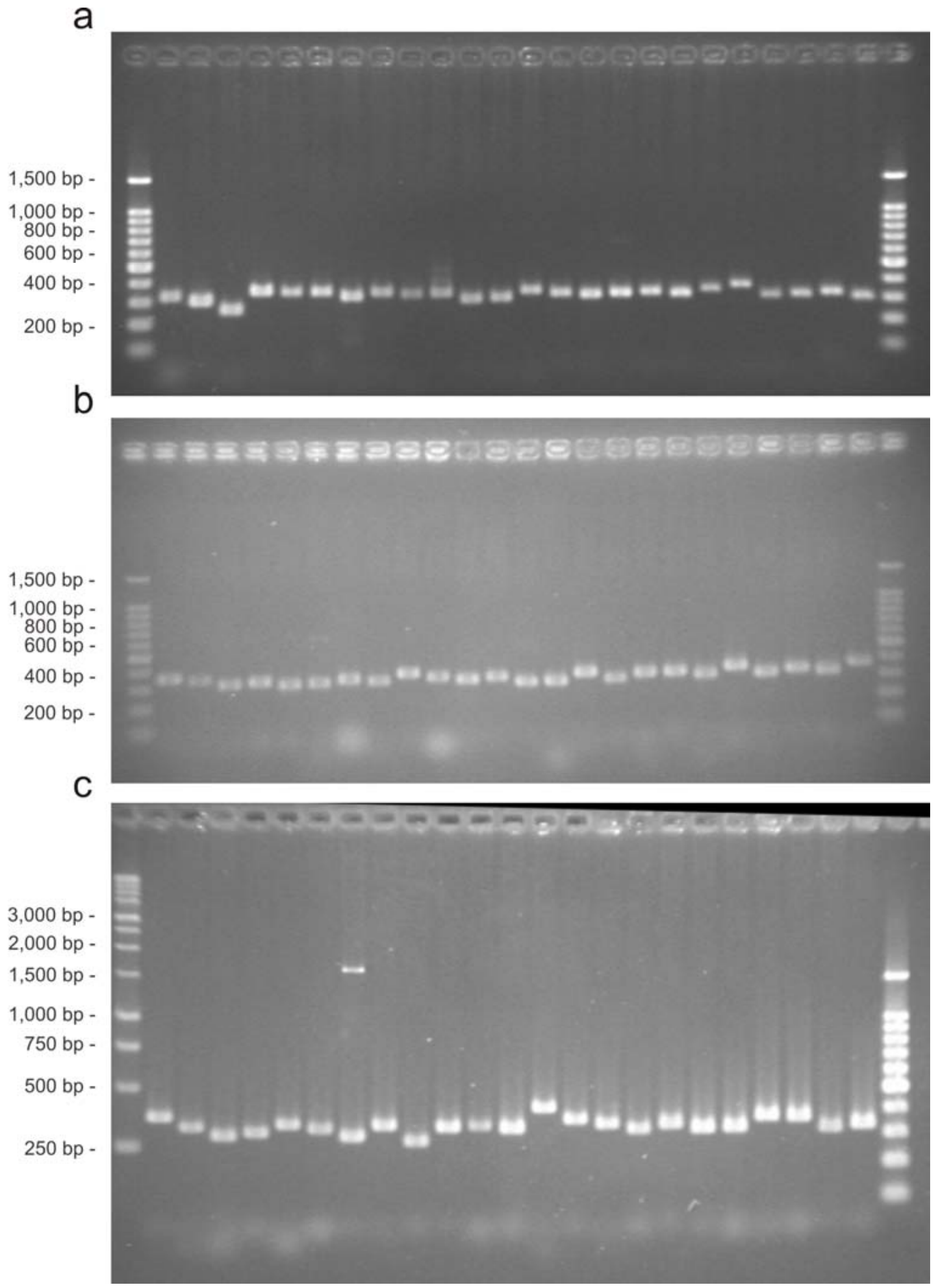

Supplementary Figure 1 | Full gel scans to Figure 3 in the main text.

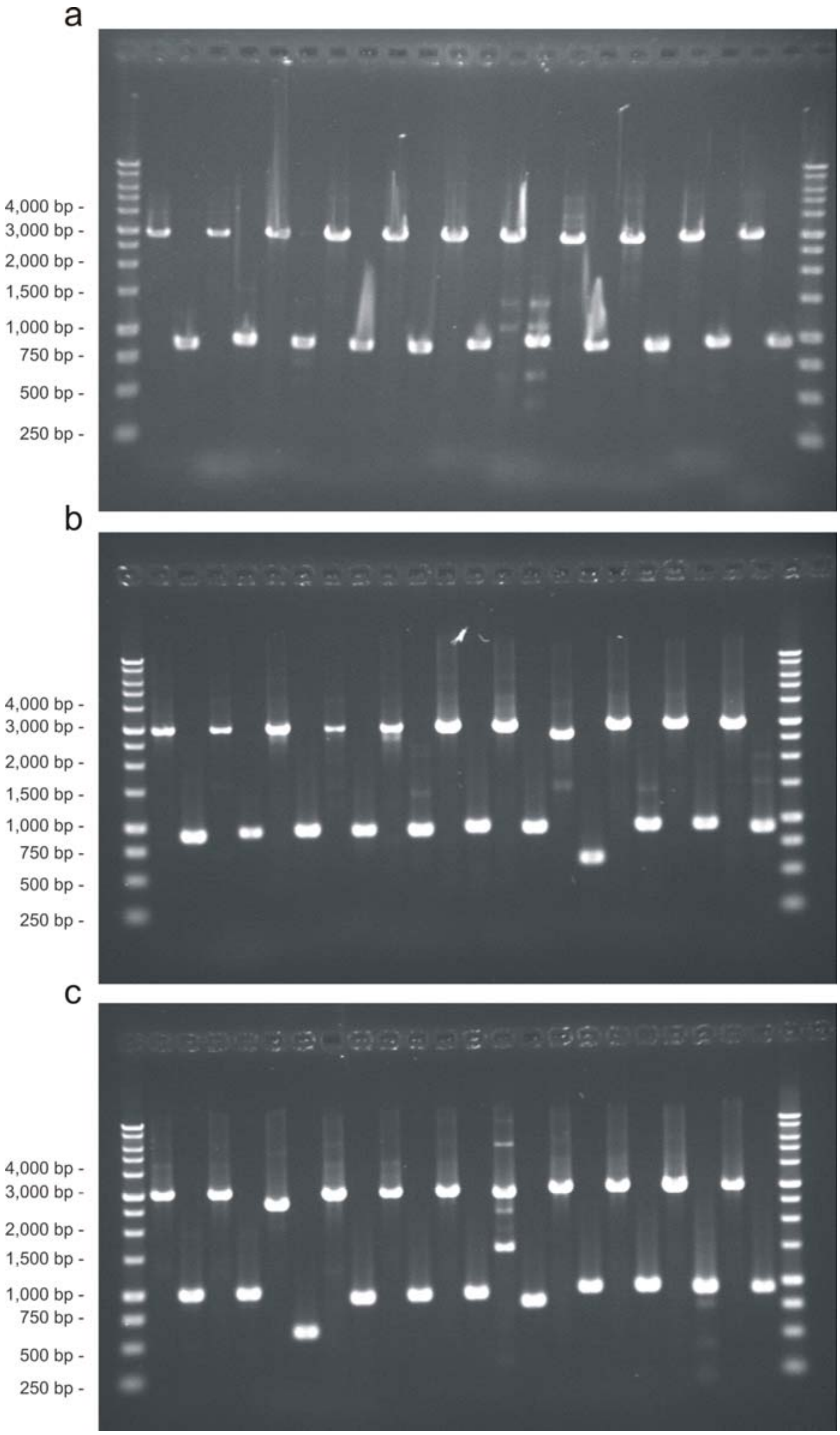

Supplementary Figure 2 | Full gel scans to Figure 4 in the main text.

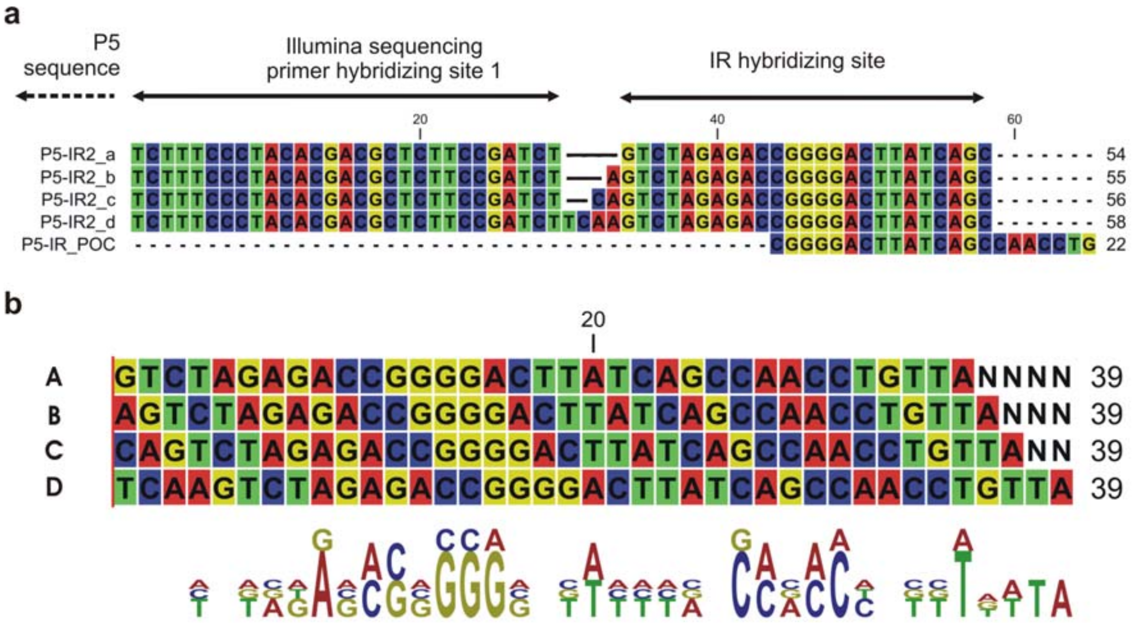

Supplementary Figure 3 | (a) Sequence alignment of the inverted repeat primers used for transposon:genomic DNA junction amplification in the complete (set of 4 primers P5-IR2 a – d) and the proof-of-concept library (P5-IR_POC). **(b)** Expected read distribution after sequencing on an Illumina sequencer. The P5-IR2 primer set creates a degenerated base read output after sequencing with the standard Illumina sequencing primer.

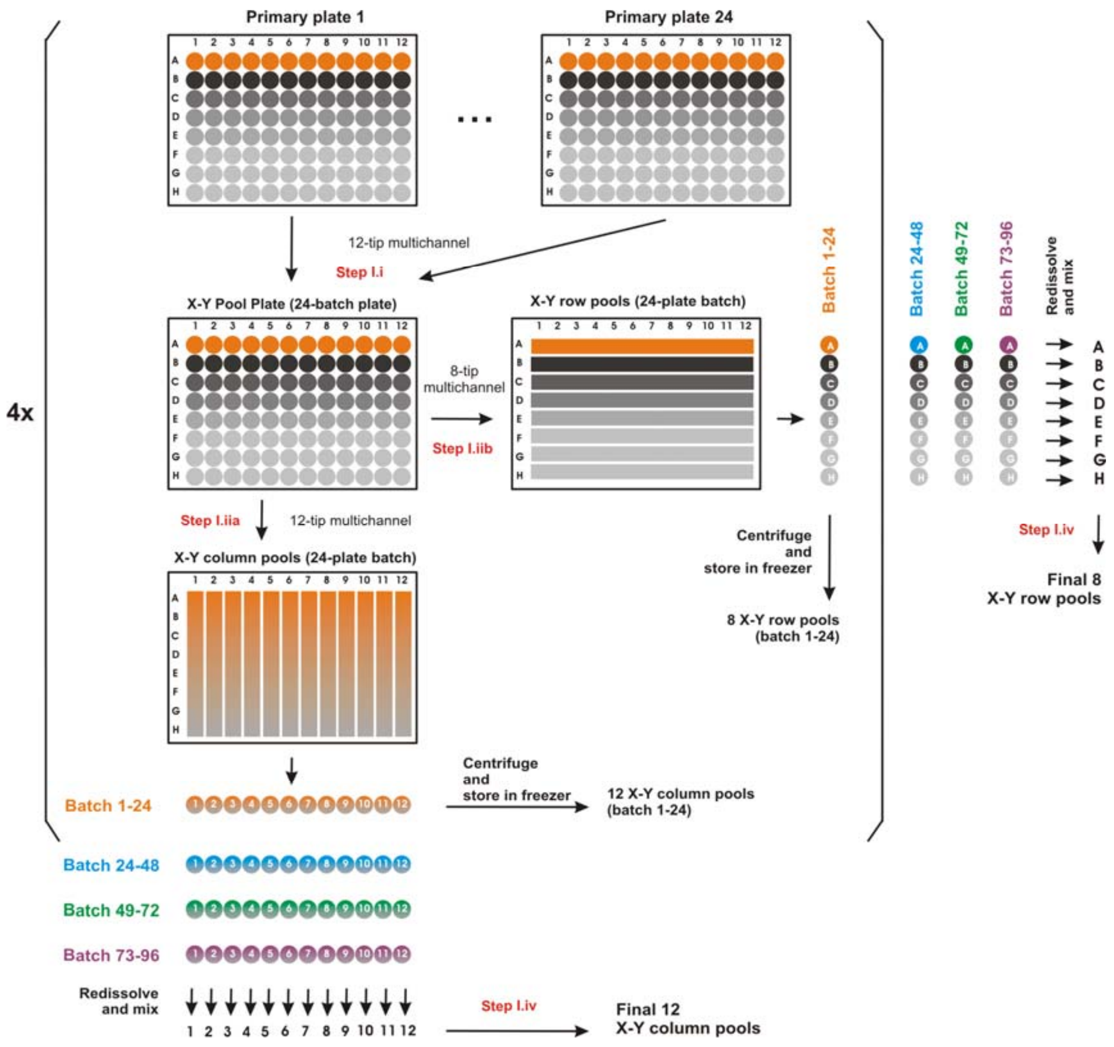

Supplementary Figure 4 | Practical implementation of Cartesian Pooling using a standard multi-channel pipette: X-Y Plate Pooling.

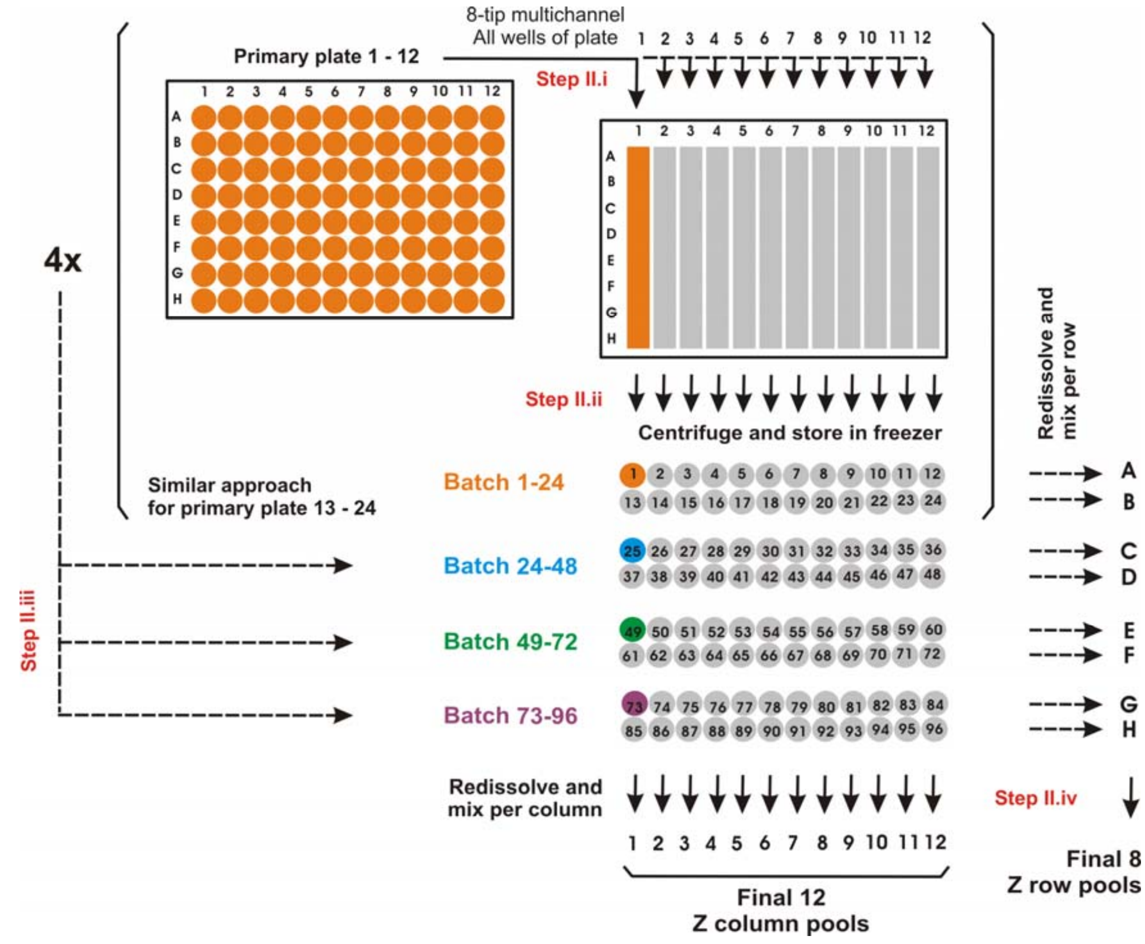

Supplementary Figure 5 | Practical implementation of Cartesian Pooling using a standard multi-channel pipette: Z Plate Pooling.

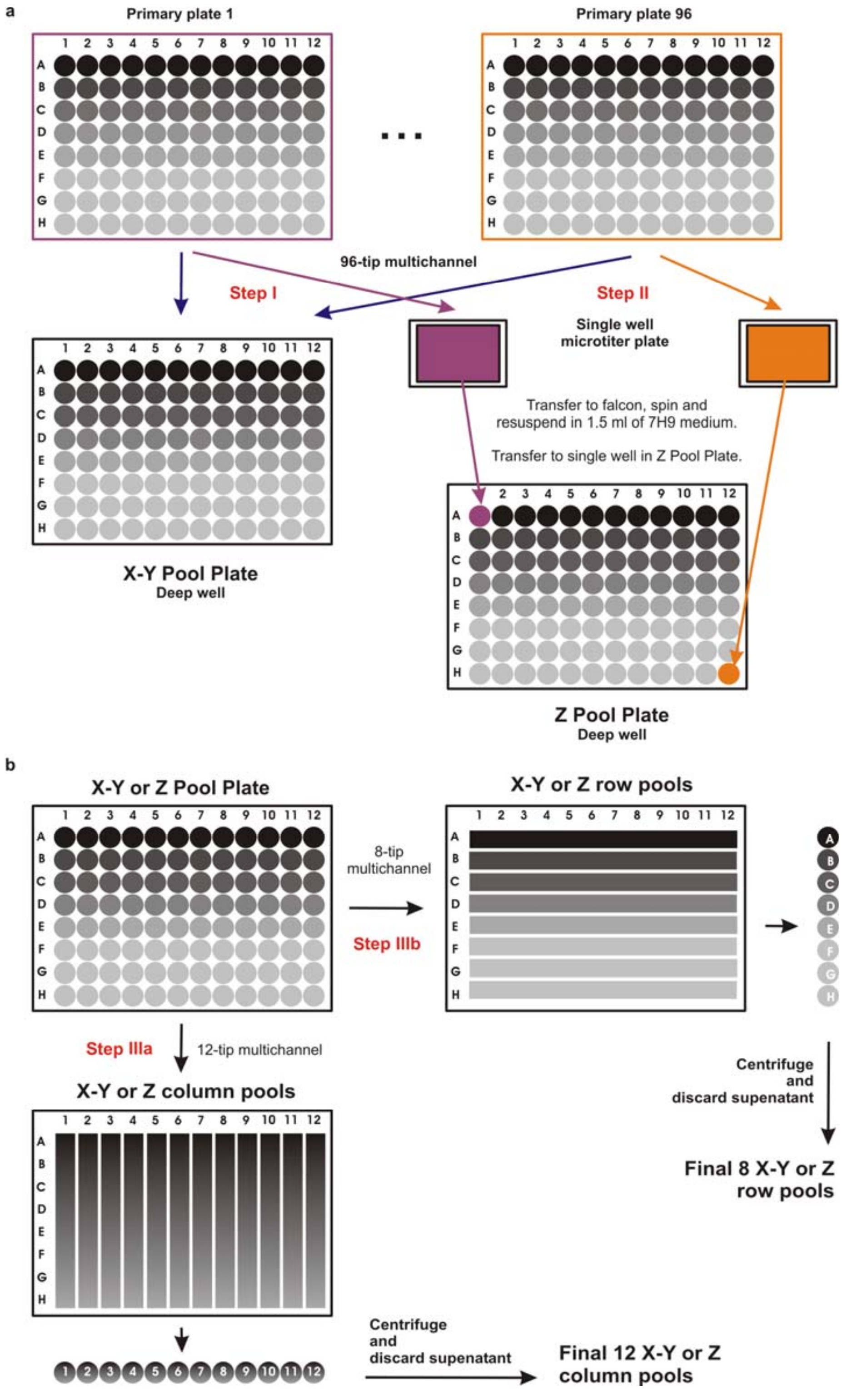

Supplementary Figure 6 | Practical implementation of Cartesian Pooling using a 96-tip multichannel pipette.

Supplementary Note 1 – Optimization of the library preparation protocol

A customized library preparation approach based on the TraDIS (Transposon-directed insertion-site sequencing) protocol[1] was optimized during proof-of-concept experiments using a subsection of the library (9 out of 96 plates). Here, genomic DNA of the mutant pools was partially digested separately with *Hin*PI and *Msp*I, purified and adaptor ligated as described before[2]. A minor modification to this previously described protocol was made by adding an 8 bp barcode (different for each pool) and the standard Illumina index sequencing primer site to the adaptor **(Supplementary Data 2)**. This was followed by a nested PCR (Phusion polymerase, Finnzymes) with primer P5-IR (binds the transposon inverted repeat and contains the Illumina P5 overhang) and the P7 primer as described[3]. The resulting PCR products were combined, size selected (± 500 bp), purified and sequenced with a custom sequencing primer on an Illumina MiSeq device.

In particular, for gDNA fragmentation we found that shearing by sonication yielded much more equal sequencing coverage of both Tn-flanking sequences than restriction enzyme fragmentation. This was measured by the ratio of forward and reverse reads overlapping at the TA dinucleotides. If the fragmentation occurs completely random, and without any PCR bias at later steps, we would expect an equal ratio of up- and downstream Tn adjacent sequences as the inverted repeat (IR) primer should bind both transposon ends equally well. Shearing by sonication creates a ratio of up- and downstream Tn junctions with an average value of 2.52 (SD ± 40.26), while this is much higher after fragmentation by RE digestion (23.55 ± 136.33).

A second set of optimizations was made at the P5-Inverted repeat (IR) primer that was designed for PCR-based amplification of Tn:gDNA junctions as well as for Illumina sequencing. Whereas we first sequenced with a custom sequencing primer hybridizing to the inverted repeat, we subsequently incorporated the standard Illumina sequencing site in the PCR amplicons that cover the Tn:gDNa junctions. This yielded an overall better quality of the sequencing data (average phred score of 35 versus 30). We also shifted the annealing site of the IR primer a few bp upstream from the transposon end, creating an 8 bp Tn-specific tag in sequencing reads that truly derive from the Tn:gDNA junctions. This greatly increased our ability to identify sequencing reads mapping to true insertion events.

Furthermore, all sequencing reads start with an identical stretch of sequence on the entire sequencing lane, which precludes the automated in-experiment instrument base-calling calibration. To compensate for this, high amounts of irrelevant (PhiX) amplicons need to be added to the chip, which very strongly reduces the sequencing yield of the experiment. To overcome this problem, we created a set of four P5-IR primers, varying both in the number and identity of the bases between the standard Illumina sequencing site and the specific 3' region that hybridizes to the transposon inverted repeat (**Supplementary Fig. 3**). This results in the

detection of the four different bases in roughly equal proportion during the first cycles of Illumina base-calling, allowing for the basecalling calibration.

Supplementary Methods

Detailed protocol for the Cartesian Pooling-Coordinate Sequencing approach for *Mycobacterium* transposon-tagged mutant libraries

This protocol outlines the detailed CP-CSeq procedure described in our article, with an emphasis on the Cartesian pooling steps and the subsequent custom Illumina library preparation and sequencing data analysis. The mycobacterial transposon mutagenesis procedure is extensively described elsewhere[4].

MUTANT LIBRARY CONSTRUCTION AND LIBRARY CARTESIAN-POOLING

SPECIAL EQUIPMENT

- Laminar flow cabinets
- BSL1-3 facility (depending on the organism of interest)
- 37°C incubator (preferably with humidity control option)
- Multichannel pipette
- Optional: 96-well multichannel pipette

CONSUMABLES

- Filtertips
- U-bottom 96-well tissue culture plates with low evaporation lid (Falcon, Fisher Scientific catalog #08-772-17)
- 96-well deep well plates (Nunc, Sigma-Aldrich catalog #Z717266)
- 8-channel (Starlab, catalog #E2999-3208) and 12-channel multi-wells (Starlab, catalog #E2999-2112)
- Single-well plate reservoir (Starlab, catalog #2999-8696)
- Mycobacterial growth medium 7H9 (BD catalog #271310), 7H10 (BD catalog #262710), OADC (BD catalog #212351)
- Antibiotics (kanamycine (Sigma-Aldrich catalog #K4378))

1. Archived mutant library construction

I. After transposon mutagenesis, plate out the *Mycobacterium* strain of interest on suitable medium (e.g. 7H10 agar, supplemented with 10% OADC and 50 µg/ml kanamycin or other appropriate antibiotics, depending on the transposon mutagenesis phage used).

II. Once the colonies are well grown on the agar plates, inoculate single colonies into 96-well plates (U-bottom tissue culture plates with low evaporation lid (Falcon)), prefilled with 200 µl of 7H9 (supplemented with 10% OADC, 0.05% Tween-80 and appropriate antibiotics). For slow-growing mycobacteria, incubate statically for 2 – 4 weeks at 37°C.

! Picking was done manually using sterile toothpicks/tips to avoid clustered colonies. With appropriate optimization, robotic colony pickers would likely also work.

! We built a library of 96 x 96-well plates (9,216 clones), but this can be scaled up or down, with minor modifications to the subsequent steps.

! We incubated the cultures in a humidity controlled incubator to minimize evaporation.

! In our experience, mycobacterial growth is faster in smaller volumes (200 µl or less) in these U-bottom TC plates compared to higher volumes (1 ml) in deep wells. Moreover, a larger number of the mutants could be successfully cultivated in the lower-volume U-bottom wells.

III. Transfer a small volume (e.g. 20 µl) of each library plate to a fresh plate filled with medium to replicate the library. Perform the pooling steps using the original plates. Incubate the replicated plates until high cell density is reached (approximately 3 weeks for *M. bovis* BCG) and transfer to -80°C compatible storage plates (prefilled with glycerol; final concentration 20%) for long-term storage.

2. Cartesian pooling

Before starting the cartesian pooling steps, it is important to first estimate the remaining volume in the 96-well library culture plates, especially in wells at the outer rows/columns (evaporation during cultivation is strongest at the plate edges). Since the pooling of approximately equal cell amounts of each clone into the two different masterplates is the foundation of CP-CSeq, it is important that the same volume from each well is transferred to both masterplates. For example, if for a particular plate there is only 100 µl of culture left in some of the outer wells, make sure to set the multi-channel volume for the two subsequent pooling steps < 50 µl.

The Cartesian pooling concept of Figure 1 in the main text can be practically implemented in different ways, depending on available liquid handling equipment and manpower. We provide two such practical implementations here. In the implementation using a classical multi-channel pipette (option 1), 24 96-well plates can be processed in 6h by two researchers. Using a 96-channel pipette (option 2), 96 plates can be processed in a day by one person.

OPTION 1: 12-channel pipette (± 4 days, 2 persons)

I. X-Y Coordinate Pooling

I.i. First, pool 50 µl of culture of each well of primary plates 1-24 into the corresponding well of a 96-deep well masterplate (X-Y Pool Plate) using a 12-tip multichannel pipette (8 rows) (**Supplementary Fig. 4**). The positions of the wells (X and Y coordinate) remain the same.

! Make sure to use fresh tips in between changing rows to avoid well-to-well cross-contamination.

I.ii. Of this X-Y Pool Plate, create intermediate X-Y column and row pools for short-term storage in the freezer (**Supplementary Fig. 4**):

a) Transfer 3/5 of the volume of every well in a 12-channel multi-well plate using a 12-tip multichannel pipette. Transfer this first batch of column pools (1-12) into falcons/eppendorfs, centrifuge, remove supernatant and store the bacterial pellet in the freezer (X-Y Column pools Batch plate 1-24).

! Change tips in between changing rows to avoid cross-contaminations.

b) Transfer the remaining 2/5 of the volume of every well into a fresh 8-channel multi-well plate using an 8-tip multichannel. Transfer this first batch of row pools (A-H) into falcons/eppendorfs, centrifuge, remove supernatant and store the bacterial pellet in the freezer (X-Y Row pools Batch plate 1-24).

I.iii. Repeat steps I.i and I.ii for the remaining 3 batches of 24 96-well plates.

I.iv. Finally, mix together the respective pools obtained from each batch of 24-well plates as shown to the right and bottom of **Supplementary Figure 4**. In this way the final 8 X-Y row pools and 12 X-Y column pools are obtained.

II. Z Coordinate Pooling

II.i. Pool 50 µl of all wells of primary plate 1 into the same column of an intermediate 12-channel multi-well plate using an 8-tip multichannel pipette (**Supplementary Fig. 5**). In this way, all clones of primary plate 1 are pooled into column 1 of a secondary plate. Repeat this for primary plates 2-24 and use a different secondary plate column for each primary plate (12 primary plates/secondary plate).

II.ii. Of these secondary plates, create intermediate plate pools for short-term storage in the freezer by transferring the volume of every column using a single tip pipette into separate falcons/eppendorf tubes. Spin down, remove supernatant and store the bacterial pellet in the freezer (Z Column pools Batch plate 1-24).

II.iii. Repeat steps II.i and II.ii for the remaining 3 batches of 24 96-well plates.

II.iv. Redissolve and mix together these plate pools per row and per column as depicted at the bottom of **Supplementary Figure 5**.

a) Make row pools by transferring 2/5 of the volume of all pools from one row, in a new tube. Repeat for all rows to obtain the final 8 Z row pools.

b) Mix the remaining 3/5 of the volume of all pools from one column in a new tube. Repeat for all columns to obtain the final 12 Z row pools.

OPTION 2: 96-channel pipette (± 1 day, 1 person)

Using a 96-channel pipette (manual or robotic), it is possible to achieve a throughput to pool all 96 primary plates on the same day, obviating the need for intermediary steps.

I. In a first step, transfer 20 µl culture volume from each well of primary plate 1 into the corresponding well of a 96-deep well masterplate (= **X-Y Pool plate**) using a 96-channel pipette **(Supplementary Fig. 6a;** blue arrows**)**. The positions of the wells (X and Y coordinate) remain the same.

   Repeat for all 96-well plates, transferring to one and the same deep well plate.

II. In a second step (Z coordinate), transfer 20 µl of each well of primary plate 1 into a single-well microtiter-format plate (filled with 10 ml of 7H9 medium) using a 96-channel pipette **(Supplementary Fig. 6a;** orange and purple arrows**)**. Then, manually pipette this pool into a falcon tube, spin down the cells, resuspend in 1.5 ml of 7H9 medium and transfer to well A1 of the **Z Pool Plate**.

   Repeat for each of the 96 primary plates.

III. create column and row pools from these X-Y and Z Pool Plates **(Supplementary Fig. 6b)**:

   X-Y Pool Plate:

   a) First, transfer 3/5 of the volume of every well in a fresh 12-channel multi-well plate using a 12-tip multichannel. Transfer these **final 12 X-Y column pools** into falcons, centrifuge, remove supernatant and either continue with genomic DNA preparation (section 3) or store the bacterial pellet in the freezer.

   ! Change tips in between changing rows to avoid cross-contaminations.

   b) Then, transfer the remaining 2/5 of the volume of every well into a fresh 8-channel multi-well plate using an 8-tip multichannel. Transfer these **final 8 X-Y row pools** into falcons, centrifuge, remove supernatant and either continue with genomic DNA preparation (section 3) or store the bacterial pellet in the freezer.

   Z Pool Plate: Identical approach as for the X-Y Pool plate. You obtain **12 final Z column and 8 final Z row pools**.

3. Library preparation Coordinate-Sequencing

SPECIAL EQUIPMENT

- Bioruptor (Diagenode catalog # B01010001) or Covaris DNA shearing instrument
- Eppendorf magnetic stand or 96-well plate magnet (e.g. Thermo Fisher Scientific catalog #12321D or #12331D)

CONSUMABLES

- Genomic DNA preparation materials (see below)
- TPX eppendorfs for DNA shearing (Diagenode catalog #C30010010-50)
- NEBNext Blunt-end repair kit (NEB catalog #E6050S/L)
- NEBNext dA-tailing kit (NEB, catalog #E6053S/L)
- Phusion polymerase (Thermo Fisher Scientific #F-530S/L or #532S/L)
- DNA binding magnetic beads e.g. AMPure XP (Beckman Coulter, Agencourt catalog #A63880) or HighPrep PCR (GC Biotech, MagBio catalog #AC-60050)
- Custom adaptors (IDT, Supplementary Data 2)

Custom adaptors

The 40 pooled samples obtained from Cartesian pooling are subsequently processed for Illumina sequencing. Treat these samples independently during the entire library preparation protocol up until the final step. Use an aliquot of each pooled sample for library preparation and store the rest in the freezer as a back-up.

The enzymes, kits and devices mentioned below are the ones we used. Nevertheless, with minor modifications, kits from other suppliers may function equally well.

I. Genomic DNA preparation **(± 1 day, 1 person)**

Prepare genomic DNA starting from the 40 pooled bacterial pellets (see below "Preparing genomic DNA from *Mycobacterium* cells").

! Depending on the degree of saturation of the cultures, ± 2 – 10 µg of DNA can be obtained from a 2 ml culture.

II. Genomic DNA fragmentation (Bioruptor or Covaris instrument) **(± 30 min – 4 hours, depending on how many samples the device can simultaneously process)**

Transfer ± 1 µg of gDNA (20 ng/µl) into 1.5 ml TPX eppendorfs and place them in the Bioruptor device. Sonicate/shear the DNA according to the manufacturer's recommendations to reach a size range of 200 – 1000bp. Check an aliquot (e.g. 7.5 µl) of

each sample on gel to confirm that the sheared DNA ranges from ± 200 – 1,000 bp; leave the rest on ice or store in freezer for later use.

! We used the Bioruptor standard device from Diagenode. Prior to shearing your final samples, it is best to optimize the settings for your particular shearing device.

III. Blunt-end repair of the fragmented DNA (NEBNext blunt-end repair kit) (± 1 – 2 hours, including DNA clean-up)

Fragmented gDNA sample	42.5 µl
10x blunt-end repair buffer	5 µl
Enzyme mix	2.5 µl
Total	50 µl

Incubate at 20°C for 30 min.

AMPure XP DNA clean-up step. Elute in 42.5 µl of ultra-pure water.

(Optional: measure concentration of the DNA on the nanodrop and/or Qubit system.)

IV. 3’ A-tailing of blunted DNA (NEBNext dA-tailing kit) (± 1 – 2 hours, including DNA clean-up)

Blunted gDNA sample	42.5 µl
10x dA-tailing buffer	5 µl
Enzyme mix	2.5 µl
Total	50 µl

Incubate at 37°C for 30 min.

AMPure XP DNA clean-up step. Elute in 25 µl of ultra-pure water.

Measure concentration of the DNA on the nanodrop and/or Qubit system.

! Combined Blunt-end repair/dA-tailing kits exist to save time and avoid the intermediate DNA clean-up step (e.g. NEB catalog #E7442S/L).

V. Adaptor ligation (Custom adaptors, IDT) (± 1 – 2 hours, including DNA clean-up)

dA-tailed gDNA sample (30 ng/µl)	8 µl (240 ng total)
Pool-specific adaptor (10 µM)	4 µl

2x Rapid-ligation buffer	12.5 µl
Enzymatics Fast ligase	0.5 µl (300 U)
--	
Total	25 µl

Incubate at 25°C for 10 min.

AMPure XP DNA clean-up step. Elute in 20 µl of ultra-pure water.

! The adaptor and primer sequences are listed in Supplementary Data 2, tab Adaptors and tab Primers. Equimolar amounts of primer A000d and primer A00x were mixed (final concentration 10 µM), heated to 95°C for 5 min in a heating block and then slowly cooled to RT by removing the heating block from the heating device, and then further down to 4°C in a refrigerator. When stored at 4°C, these adaptors remain stable for at least several weeks.

! Add excess of adaptor to the fragmented DNA (e.g. 20 – 100 fold molar excess, e.g. using 240 ng of 200 – 1,000 bp fragments (2 – 0.4 pmol) and 4 µl of a 10 µM adaptor mix (40 pmol)).

! A single clean-up step is insufficient to completely remove the excess of unligated adaptor, although the level of removal so obtained is sufficient for allowing the subsequent PCR reaction. Presuming > 95% recovery after AMPure XP clean-up, we estimate the ligated DNA concentration at ± 10 ng/µl. For exact quantification at this step, consider multiple clean-up steps.

VI. Transposon gDNA-junction enrichment (Phusion polymerase) (± 2 - 3 hours)

5x GC buffer	10 µl
DMSO	1.5 µl
10 mM dNTPs	1 µl
P7 primer (100 µM)	0.25 µl
P5-IR2a-d primer mix (100 µM)	0.25 µl
Phusion polymerase	0.5 µl
Template (± 10 ng/µl)	5 µl
Ultra-pure water	31.5 µl
--	
Total	50 µl

Program:

98°C (3 min)

4 cyles of 98°C (20 sec), 70°C (20 sec) and 72°C (1 min)

20 cycles of 98°C (20 sec), 67°C (20 sec) and 72°C (1 min)
72°C (3 min)
12°C (until further processing)

Load an aliquot (e.g. 5 µl) of each reaction on gel to check for amplification.

! Although we only have experience with Phusion polymerase for these purposes, other high-fidelity polymerases might work equally well.

VII. **DNA pools mixing and size selection** **(± 2 - 4 hours, including DNA clean-up)**

Combine the 40 PCR reactions and run the mix on a 2% preparative agarose gel. Cut out the 250 – 750 bp region and extract the DNA from the agarose gel (e.g. Machery Nagel gel clean-up kit). Perform an extra AMPure XP DNA clean-up to make sure that the DNA is pure enough for subsequent quality control steps and finally Illumina sequencing.

! When mixing the PCR reactions, take into account that each A-H pool is derived from ± 768 mutants (96 x 8), while every 1-12 pool is derived from ± 1,152 (96 x 12) mutants. To equalize the sequencing read depth per mutant, mix 20 µl of the PCR amplificate deriving from each A-H pool vs. 30 µl of each 1-12 pool.

VIII. Confirm DNA fragment sizing on gel or on a BioAnalyzer (Agilent), measure exact DNA concentration (Nanodrop, Qubit, BioAnalyzer or qPCR-based quantification) and sequence the sample on an Illumina device using the standard Illumina sequencing primers (Single-read 100bp with barcode sequencing).

4. Sequencing data analysis and deconvolution of the mutant positions

The raw Illumina sequencing data was processed with CLC Genomics Workbench (http://www.clcbio.com/products/clc-genomics-workbench/) and the open source Galaxy platform (http://galaxyproject.org/). Although the exact sequencing data analysis pipeline may differ depending on the experimental details and the software platform used, the main analysis outline using the free Galaxy software is given below (steps I - III can also be done using the CLC Genomics Workbench). While the analysis procedure is rather straightforward, some basic knowledge of the software is required. The Galaxy tool to fulfill each operation is given in between brackets. Our optimized Galaxy workflows and the BioPerl CP-CSeq algorithm are added in **Supplementary Data 3**.

I. Raw read quality filtering and adaptor trimming

After Illumina sequencing, the Illumina software automatically demultiplexes the barcodes and generates 40 FastQ files, each one representing the raw sequencing reads of a given Cartesian pool. Import (Galaxy: Upload file) and groom (Galaxy: FastQ Groomer) the FastQ files on the Galaxy server. In addition, import the genome sequence and annotation files (for *M. bovis* BCG Pasteur strain 1173P2, Genbank accession number AM408590.1). Subsequently trim off low quality bases (Galaxy: FastQ Quality trimmer) as well as adaptor sequences (Galaxy: Clip adaptor sequences).

II. Select transposon-specific reads (Galaxy: Workflow 1 – ClipIR_TrimLength).

The workflow selects only those reads containing the 8 bp transposon-specific tag (CAACCTGT) and discards the rest. Next, it trims the remaining reads to a given length (set standard to 25bp, which is sufficient to confidently map reads to a bacterial genome).

! This trimming step facilitates the calculation of the coverage of each mutant (at their inserted TA in the genome) at later steps.

III. Read mapping to the reference genome

Map the reads to the organism's reference genome (Galaxy: Map with Bowtie for Illumina). This will generate a SAM/BAM output for each Cartesian pool (= alignment file with mapping information for each read).

! Discard reads that do not uniquely map to the reference genome. This avoids the uncertain mapping of mutants in duplicated regions of the genome.

IV. Generate the transposon-insertion mutant list

To generate a list of every transposon-insertion mutant present in the library, first merge all BAM files of all Cartesian coordinate groups together (Galaxy: Merge BAM files) and convert this merged file to the SAM file format (Galaxy: BAM-to-SAM). From this SAM file, generate an independent file containing the coverage at each TA dinucleotide with reads that map to the top strand of the genome, and another one for coverage with reads that map to the bottom strand (Galaxy: Workflow 2a_ForwardReads and Workflow 2b_ReverseReads). Join these independent files to create a complete transposon-insertion list of the entire library, discarding TA's where only forward or reverse reads start (Galaxy: Workflow 3_CreateTnList_FwRevReads). This generates a BED file ('TnList') containing the position in the genome of each TA in which a transposon is inserted.

! Additional information can be added to this transposon-insertion mutant BED file e.g. adding gene information requires a genome's annotation file (Galaxy: Join the intervals of two datasets side-by-side).

V. Calculate coverage file for each Cartesian pool

Run above workflows (Galaxy Workflow 2a, 2b and 3) for each Cartesian pool SAM file. This generates a BED file for each pool, containing coverage information of each mutant in that pool. Next, paste these coverage files together per Cartesian pool group (A-H and 1-12 for both X-Y and Z Pool Plates) (Galaxy: Workflow 4a – Join8Datasets_A-H and Workflow 4b – Join12Datasets_1-12). These BED files (one mutant per row, Cartesian pool coverage per column) are in tab-delimited format and can then be further manipulated in e.g. MS Excel. Combine both X-Y BED files (1-12 and A-H) together, thus creating a tab-delimited file with first 7 columns (column c1 to c7) mutant and gene information, and then 20 columns containing the reads counts of each mutant per Cartesian pool (pool 1-12 in c8 to c19, pool A-H in c21 to c28; c20 is empty). Do the same for the Z BED files. Save files respectively as 'TnList_XY.txt' and 'TnList_Z.txt'.

! The provided Galaxy Workflows 4a and 4b require 7 columns in the input 'TnList' BED file from step IV (with data columns c2 and c3 = TA start and end position).

! Normalize each read count in these files to the average number of reads per Cartesian pool group. E.g. if the total number of obtained sequencing reads from the Illumina software in Cartesian pool X-Y column 1 is 2.2 million, and the average number across column pool 1 to 12 is 2 million, divide every read count in X-Y column 1 by a factor of 1.1 (2.2 million over 2 million).

VI. CP-CSeq algorithm

Run the CP-CSeq algorithm (CP-CSeq_BioPerlScript), which generates an output file with coordinates for each mutant (= row) in the list, provided that sufficient data for that mutant is present in the dataset.

! The algorithm requires the two tabular (.txt) input files ('TnList_XY.txt' and 'TnList_Z.txt') from step V, each containing the 7 initial columns from the TnList file of step IV (c2 and c3 = TA start and end position, c4 = unique mutant identifier) and the 20 additional read count columns (c8 to c19 = pool 1-12, c21 to c28 = pool A-H; c20 = empty) added in step V.

VII. Visualisation

Visualize the SAM files of each Cartesian Pool, generated in step III, using a genome browser or analytical software package (we used CLC Genome Workbench). This allows to visually cross-check the CP-CSeq algorithm's output for the mutants of interest to a particular project, prior to starting the work of taking these mutants out of the library for further characterization.

Preparing genomic DNA from *Mycobacterium* cells

CONSUMABLES

- **Lysozyme** (Sigma catalog #L6876)
  Prepare 5 mg/ml lysozyme solution in 20 mM Tris/HCl pH 9; Store in small aliquots at -20°C (do not repeatedly freeze and thaw).
- **SDS** solution (10%; store at RT)
- **Proteïnase K** (2 mg/ml; store aliquots at -20°C)
- **NaCl** (5 M)
- **CTAB/NaCl** (10% CTAB in 0.7M NaCl)
  Dissolve 4.1 g of NaCl in 80 ml of distilled water. While stirring, add 10 g of CTAB. If necessary, heat solution to 65°C. Adjust volume to 100 ml with distilled water. Store at RT for no longer than 6 months.
- **Phenol/Chloroform/Isoamyl alcohol** (Thermo Fisher Scientific catalog #15593-031; store at 4°C or at -20°C)
- **Isopropanol**
- **75% Ethanol**
- **Ultra-pure water**

PROTOCOL

- Add 100µl of 5mg/ml lysozyme to the mycobacterial pellet and mix gently.
- Incubate O/N at 37°C (shaking).
- Add 10% SDS to a final concentration of 2% (30µl) and Proteïnase K to a final concentration of 33µg/ml (2.5µl).
  Adjust the volume to 150µl with 20mM Tris/HCl pH 9 (+17.5µl).
- Incubate for 3h at 37°C (shaking).
- Add 30µl of 5M NaCl.
- Add 30µl of CTAB/NaCl which is prewarmed at 65°C.
- Vortex and incubate 15 minutes at 65°C.
- Add 200µl of Phenol/Chloroform and mix gently by inverting the tube a few times.
  Centrifuge at RT for 15 minutes at 11,000xg.
  Transfer aqueous supernatant to a new tube.
- Add the same volume of Phenol/Chloroform, mix gently by inverting the tube a few times.
  Centrifuge at RT for 10 minutes at 13,000xg.
  Transfer aqueous supernatant to a new tube.
- Add 0.6 volumes of isopropanol to precipitate the nucleic acids.
  Place at least 30 minutes at -20°C (or longer).
  Centrifuge at RT for 10 minutes at 13,000xg, discard the supernatant.
- Add 1ml of cold 75% Ethanol and invert the tube a few times.
  Centrifuge at RT for 10 minutes at 13,000xg, remove supernatant carefully.
  Dry the pellet (10 minutes).
- Resuspend the pellet in 30µl of ultra-pure water (O/N at 4°C).
- Measure the DNA concentration spectrophotometrically (e.g. on a Nanodrop instrument).

Supplementary references

1. Langridge, G. C. *et al.* Simultaneous assay of every *Salmonella Typhi* gene using one million transposon mutants. *Genome Res.* **19,** 2308–2316 (2009).
2. Sassetti, C. M., Boyd, D. H. & Rubin, E. J. Comprehensive identification of conditionally essential genes in mycobacteria. *Proc Natl Acad Sci U S A.* **98,** 12712–12717 (2001).
3. Griffin, J. E. *et al.* High-resolution phenotypic profiling defines genes essential for mycobacterial growth and cholesterol catabolism. *PLoS Pathog.* **7,** e1002251 (2011).
4. Murry, J. P., Sassetti, C. M., Lane, J. M., Xie, Z. & Rubin, E. J. Transposon site hybridization in *Mycobacterium tuberculosis*. *Methods Mol Biol.* **416,** 45–59 (2008).
